# Antibiotics utilization and farmers’ knowledge of its effects on soil ecosystem in the coastal drylands of Ghana

**DOI:** 10.1371/journal.pone.0228777

**Published:** 2020-02-06

**Authors:** Christian Adler Phares, Andrews Danquah, Kofi Atiah, Frimpong Kwame Agyei, Osei-Tutu Michael

**Affiliations:** 1 Department of Soil Science, University of Cape Coast, Cape Coast, Ghana; 2 Department of Molecular Biology and Biotechnology, University of Cape Coast, Cape Coast, Ghana; Tokat Gaziosmanpasa University, TURKEY

## Abstract

**Background:**

There is paucity of information on antibiotics utilization amongst farmers, factors associated with administration of antibiotics and farmers’ knowledge of the effects of antibiotics on the soil ecosystem in Ghana.

**Methods:**

A cross sectional quantitative survey across three coastal regions of Ghana was undertaken amongst poultry and livestock farmers. Six hundred respondents were selected from five districts each across the three regions. Pretested and structured questionnaire were used to collect data through face to face interview. Data were summarized using descriptive statistics and regression analysis. Factors associated with antibiotic administration were determined using binary multiple logistic regression at p ≤ 0.05.

**Results:**

Out of the 600 farmers, 95% administered antibiotics and 84% bought antibiotics over-the-counter without prescription. Approximately 9% of antibiotic administration was carried out by veterinary officers, and the remaining, 91% based on farmer’s experience. Approximately 93% had access to antibiotics without any difficulty. Withdrawal period was always observed by only 16% of farmers. Majority (74%) of farmers never had education on antibiotics and none of the farmers screened manure for the antibiotic residuals. Years of farming, income status, level of education of farmers, type of animal kept, access to extension services, registration with farmers’ association, employing veterinary services, location of farm, system of production, education on antibiotics and access to antibiotics positively and significantly predicted the administration of antibiotics by farmers. Majority of farmers had inadequate knowledge of the effects of antibiotics on soil ecosystem with mean score ranging between 2.87±0.60 and 2.98 ± 0.7 on a scale of 5.0.

**Conclusion:**

The study exposed the poor practices regarding antibiotic use and also inadequate knowledge on its effect on the soil ecosystem amongst farmers in Ghana. This calls for development of strategies to increase awareness on antibiotics because its misuse can negatively impact human, animals, environment and impact food security.

## Background

Antibiotic use is widespread not only in the general population but also in the agricultural industry for crop and animal production. Substantial evidence exists on successful use of wide range of these antibiotics in farm animals for metaphylaxis, prophylaxis and growth promotion [1, 2, 3]. This results in the production of healthy meat from livestock, by virtue of increase in infection prevention and outbreaks as well as growth promotion. Antibiotics have also been indispensable for crop production, having been used in this sector for more than 50 years in the United States [4]. Consequently, significant residues of veterinary antibiotics have been reported in crop field and harvested crops [5, 6].

In the pig, dairy and poultry industries, antibiotics are used extensively, as component of their feeding and routine management practices globally, either to prevent diseases or stimulate growth [7, 8]. The manure, from these animals has been reported to contain residuals of antibiotics fed to them [9, 10]. Due to the transition of antibiotic through the animal body systems, there is a tendency of reduction in the lethal dose of the antibiotics that ends up in the manure. Animal’s dropping and urine contain between 70%–90% of the antibiotic administered unchanged or in active metabolites [11]. Subsequently, the antibiotics contained in the manure would have low efficacy to kill bacteria they come in contact with, potentially predisposing the exposed bacteria to resistance gene development. In some instances, the manure may also carry resistance genes [12, 13] which are introduced into farms when these manures are applied as soil amendments. The emerged resistance genes and resistant bacteria are disseminated directly through soils, ground and surface water, atmosphere, contaminated crops to healthy animals and humans.

Over the years, growing concerns from the public and the scientific community have been consistent in relation to the emergence and/or development of microbial resistance from exposure to clinical and veterinary antibiotics. These are legitimate concerns because of the potential negative impact antibiotics could have on humans, soil health and subsequently food security. A number of disease conditions have been reported in humans [14, 15], livestock and plant that were traced to antimicrobial resistance genes. Besides, human exposure to these resistant microbes and genes could result in human and animal mortality. It is estimated that more than 2 million Americans are infected with antibiotic resistant bacteria annually, out of which 23,000 died. About 10 million deaths have been predicted to occur as a result of exposure to antibiotic resistance microbes [16]. Moreover, exposure to resistance microbes could result in the loss of significant number of working days due to indisposition and time required to treat infection. Additionally, the sick is burdened financially through the cost involved in infection treatment. Control of plant diseases caused by resistance microbes could be difficult, affecting yield and subsequently food security.

In soil, antibiotics could negatively impact soil health through its interaction with the microbial community. It has been reported that antibiotics affect the overall population of soil microorganisms [17, 18], enzyme activity [19, 20], carbon and nitrogen mineralization [21], and competition with cations for exchange sites [22].

In order to regulate antibiotic use, conserve its efficacy and reduce or prevent the development of microbial selection in the environment and prevent disruption of soil biodiversity, the World Health Organization (WHO) has suggested its use must be under supervision of licensed veterinary officer and related health experts [23]. In addition, pragmatic efforts are required by stakeholders both in medical practice and agriculture to ensure ‘best practices’ which include but not limited to, buying and dispensing of antibiotics under official prescriptions, administration of antibiotics to animals at the oversight of veterinarian and observing withdrawal periods.

Farmers are major stakeholders in antibiotic utilization, yet there is paucity of information regarding the extent to which they utilize antibiotics and their characteristics that influence the administration of antibiotics in Ghana. Few studies conducted have not ascertained farmers’ knowledge on what happens to soil ecosystem when antibiotics are applied to soil [24, 25]. This research was conducted to assess the extent of utilization and practices regarding antibiotics amongst farmers. The second objective determined factors associated with the administration of antibiotics and the last, assessed farmers’ knowledge of the effects of antibiotics on the soil ecosystem. The findings of our study could be incorporated into intervention protocols that seek to educate farmers and the public regarding antibiotics.

## Materials and methods

### Study design, setting and participants

A cross sectional method was adopted for the study between September, 2018 and April, 2019 in three Coastal regions of Ghana; Volta, Central and Greater Accra. Farmers in those regions are greatly involved in the raising of poultry, pigs and diary yet there is limited information on their antibiotics utilization. The three regions have 25 (Volta), 20 (Central) and 26 (Greater Accra) districts and have farmers who are greatly involved in poultry and livestock production (personal communication with District Directors of the Ministry of Food and Agriculture). The directors opined that poultry and livestock are a major part of Ghanaian traditional meals serving the protein needs in most household diets hence their production is increasing each day. The manure is used as fertilizers for crop production [26] probably because they come at no cost to the farmer and are readily available in most farms where poultry and livestock are raised.

### Sampling

**Fig 1:**
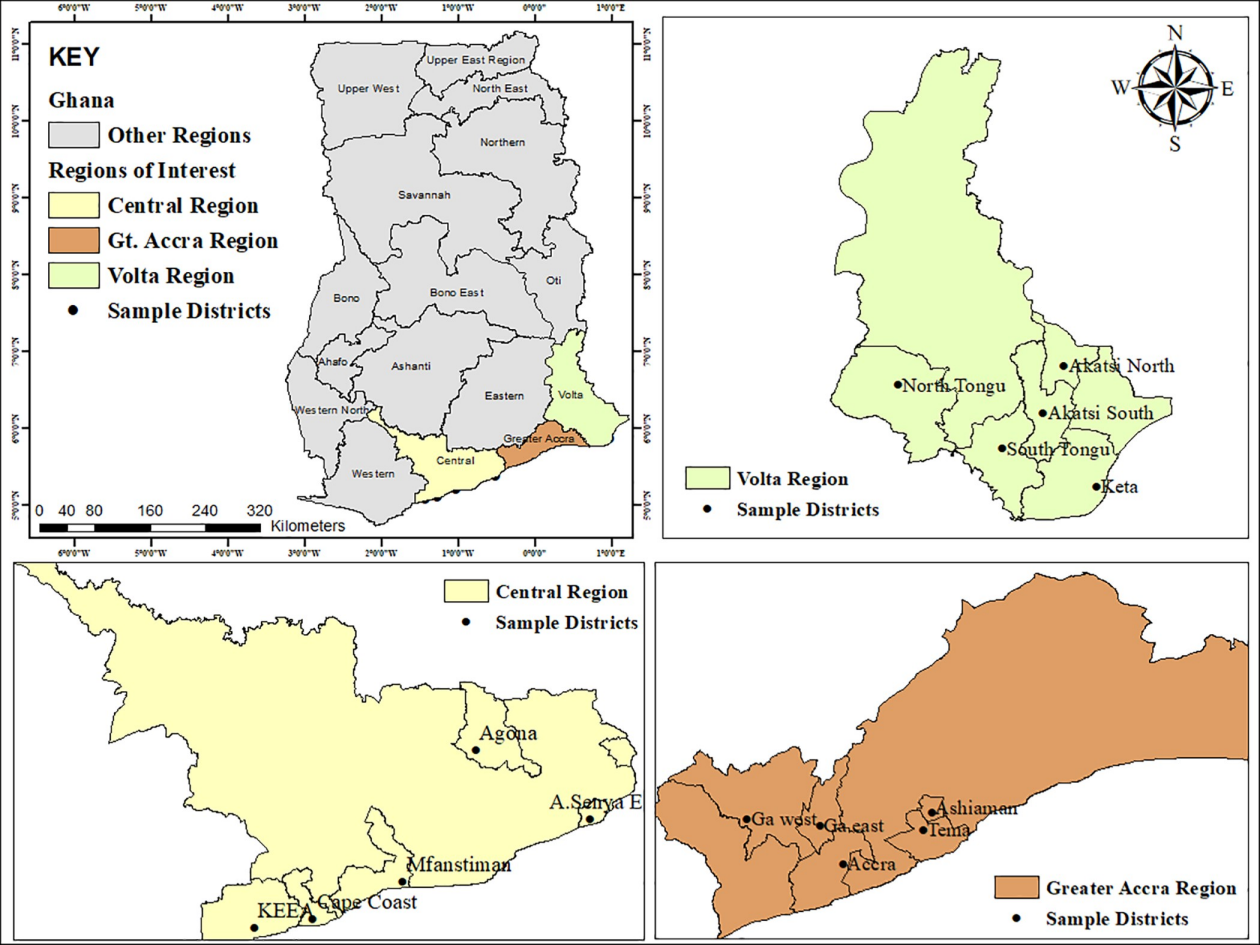
Map of Ghana showing study regions (Central, Volta and Greater Accra regions) and name of sample districts.

The study employed multistage sampling technique. Five districts from each region (Fig 1) were randomly selected for the study employing the lottery approach. The names of the districts were written on pieces of paper, placed in a container and drawn one at a time until five districts each were selected. The lottery approach ensured all the districts had equal chance of being drawn for the study. Within each selected district, farmers who raised poultry, pigs and cattle and, where manure droppings from these farms are utilized as fertilizers for crop production, met the inclusive criteria for the study.

Farmers who met the criteria were listed to participate in the study. A total of 702 farmers were listed but only 600, representing 85%, agreed to enroll on the study. The 15% who could not participate was due to time unavailability, accessibility to farms and small farm sizes. Quantitative survey of 600 farmers who agreed to participate in the study were undertaken.

### Data collection

An initial meeting was scheduled with the farmers who met the eligibility criteria. A total of six data collectors and two supervisors were trained to assist in data collection. The training provided the data collection assistants information on: aim of the study, reasons for confidentiality of respondent’s information, rights of respondents and data collection procedure.

Data collection was done using a pretested and structured questionnaire. The questionnaire had three sections based on the objectives of the study. The sections included; farmers’ socio demographic features and other farm characteristics, practices regarding antibiotics utilization and knowledge of farmers on the effects of antibiotics on the soil ecosystem. Variables about farmers and farm characteristics captured were: sex, age, years of farming, level of education, income, type of animal kept, location of farm, system of production. The second section solicited information on whether farmers had ever administered antibiotics. Farmers who responded in the affirmative were interrogated on where they bought the antibiotics, who administered the antibiotics, whether dosage administered was recommended or it was based on farmers’ experience and reasons of given antibiotics to their animals. Other farmer’s characteristics interrogated were: access to extension services, access to veterinary services, access to antibiotics, education on antibiotics, observation of withdrawal periods and screening of manure for antibiotics presence and quantity. The last section solicited information on the knowledge of farmers regarding the effect of antibiotics on soil ecosystem. The section had seven items and responses were on a five point Likert scale in a continuum from “not sure” to “strongly agree’ coded as 1 = strongly disagree, 2 = disagree, 3 = not sure, 4 = agree and 5 = strongly agree. The items were; ‘substantial quantity of antibiotic given to animals end up in manure’, ‘antibiotics compete with other soil cations/anions for exchange site’, ‘antibiotics cause the death/inhibit growth of microbial groups involved in ecosystem functions’, ‘antibiotics influence nutrients transformation; decrease nitrification in N cycle’, ‘antibiotics cause decrease in bacteria/fungi ratio in soil’, ‘antibiotic resistance genes (ARGs) in soil increase the likelihood of human pathogens acquiring resistance’, and ‘antibiotics decrease soil respiration’.

Prior to data collection, the questionnaire was piloted on 15 farmers, which prompted some minor modification to suit the research objectives. Data were collected by explaining the questionnaire to the respondents in English. However, the dominant native language ‘Twi’ and ‘Ewe” were used where farmers had no formal education or could not read and write.

### Data analysis

Descriptive statistics including mean and standard deviation as well as inferential statistics was employed to summarize the data. Chi square association between set of explanatory variables (farm and farmer’s characteristics) and the dependent variable (antibiotic administration) was established at 95% confidence interval. Additionally, Cramer’s V was used to determine the magnitude of association between variables. Variables that had a significant positive relationship analyzed using Chi square, were considered in a binary multiple logistic regression to establish the relative crude and adjusted odd ratios at 95% confidence interval. Multicollinearity among independent variables was checked using variance inflation factor (VIF) and goodness of fit by Hosmer and Lemeshow respectively. Mean score rating for knowledge of farmers was done with the following interpretation; 1.0–1.9 = ‘very poor knowledge’, 2.0–2.9 = ‘poor knowledge’, 3.0–3.9 = ‘good knowledge’, > 3.9 = ‘very good knowledge’. The mean rating was translated from the mean analysis of the five point Likert scale used to determine the extent of farmers’ knowledge on antibiotic effects on soil ecosystem.

### Ethical statement

The study was reviewed and approved by Authors, and an internal Research Board of the School of Agriculture, University of Cape Coast. The aim of the study was explained to the participants in their mother tongue. ‘Twi’ was used for participants from Central and Greater Accra regions and ‘Ewe’ for those from Volta region for easy understanding since appreciable number of the farmers could not read, write and comprehend the English language. Informed permission was obtained from the respondents before questionnaires were administered and they were told that their identification was not required. Furthermore, they were told they could opt out from the study at any time. The consent was verbal due to the fact that their responses do not have any personal, economic and or political ramifications. Moreover, no risk was identified to be associated with the responses that could endanger the lives of the participants. The confidentiality of solicited information from the participants was ensured and access to answered questionnaire was permitted after agreement was reached by the researchers.

## Results

### Farmers and farm characteristics

A total of 600 respondents enrolled on the study. Table 1 present farmers and farm characteristics. Majority (71%) of the respondents were males and 29% were female. The age range was between 21 and 63 with majority (80.5%) greater than 30. About 69% were married, with the rest unmarried. Slightly above half (57.0%) of the farmers had been in the animal rearing enterprise for less than 10 years and 43% had worked in the industry for 10 years or more. Most (80%) of the farmers were in the low income bracket and appreciable number (31%) being illiterates. Of the 600 farmers’ majority (70%) raised poultry and the remaining were into other livestock enterprise. Regarding access to extension services, 66.5% responded in the affirmative of having access to extension services and about 60% of the 600 farmers had registered with farmers’ association. Approximately, 66.2% of the farmers were located in the urban communities, whiles 33.8% were located in the rural/peri urban areas, respectively. Greater percentage (72%) practiced controlled system, and the remaining 28%, used uncontrolled animal production systems. In addition, 35% indicated having had access to veterinary officers with the majority (65%) never having access. On the issue of education on antibiotics, majority representing 73.8% responded in the negative of been educated on antibiotics. Meanwhile, higher percentage of 92.8% responded in the positive that access to antibiotics was easy. Results of the study showed that sex, age and marital status of farmers were not associated with antibiotics utilization. However, years of farming, income status, level of education, type of animal kept, access to extension services, registration with farmer’s groups, access to veterinary services, location of farm, system of production, education on antibiotics, and access to antibiotics showed significant relationship with antibiotic utilization. Cramer’s V results indicated positive but weak relationship with antibiotic utilization.

**Table 1:** Farmers and farm characteristics (N = 600).

Farm/Farm characteristics	Categories	Administered antibiotics		Cramers V	X^2^	*P* value
	F (%)	Yes No				
**Sex**				0.02	0.24	0.63
Male	427 (71.2)	403	24			
Female	173 (28.8)	165	8			
**Age**				0.05	1.60	0.21
< 30 years	117 (19.5)	108	9			
30 years and more	483 (80.5)	460	23			
**Marital status**				0.03	0.63	0.43
Married	413 (68.8)	393	20			
Unmarried	187 (31.2)	175	12			
**Years of farming**				0.12	9.15	<0.01
less than 10 years	342 (57.0)	332	10			
10 years and above	258 (43)	236	22			
**Income status**				0.10	6.02	0.01
High	120 (20.0)	119	1			
Low	480 (80.0)	449	31			
**Level of education**				0.09	5.33	0.02
Illiterate	185 (30.8)	181	4			
Literate	415 (69.2)	387	28			
**Type of animal kept**				0.12	8.76	<0.01
Poultry	421 (70.2)	406	15			
Livestock	179 (29.8)	162	17			
**Access to extension services **				0.09	5.84	0.02
Yes	399 (66.5)	384	15			
No	201 (33.5)	184	17			
**Registered with farmers association**				0.18	20.1	<0.001
Yes	358 (59.7)	351	7			
No	242 (40.3)	217	25			
**Employed veterinary services**				0.17	16.9	<0.001
Yes	210 (35.0)	188	22			
No	390 (65.0)	380	10			
**Location of farm**				0.19	21.9	<0.001
Urban	397 (66.2)	388	9			
Rural/Peri Urban	203 (33.8)	180	23			
**System of production**				0.25	37.04	<0.001
Controlled	432 (72.0)	424	8			
Uncontrolled	168 (28.0)	144	24			
**Education on antibiotic effects**				0.09	4.93	0.03
Yes	157 (26.2)	154	3			
No	443 (73.8)	414	29			
**Access to antibiotics**				0.11	6.82	0.09
Easy	557 (92.8)	531	26			
Difficult	43 (7.2)	37	6			

Frequency (F) and Percent (%) of participant’s responses, N = total number of respondents

### Farmer’s practices involving antibiotic use

Table 2 show practices regarding utilization of antibiotics by farmers. Antibiotics was administered by 94.7% of the 600 farmers with only few (5.3%) indicating otherwise. Majority (86.3%) of farmers who gave antibiotics did that to prevent and treat diseases while 13.0% said it was done to promote growth. On how farmers get access to antibiotics, the results showed that majority (84.2%), bought it over-the-counter without prescription, 10.0% bought with veterinary prescription and 3.9% from agro dealers without prescription and 1.9% from colleague farmers without prescription.

**Table 2:** Practices regarding antibiotics utilization amongst farmers.

Administration of antibiotics (N = 600)	Frequency	%
Yes	568.0	94.7
**Where do you buy the antibiotics? (N = 568)**		
Purchased with veterinary prescription	57.0	10.0
Purchased over the counter without prescription	478.0	84.2
Colleague farmer without prescription	11.0	1.9
Agro dealer without prescription	22.0	3.9
**Who administered antibiotics (N = 568)**		
Veterinary officer	48.0	8.5
Farm manager	132.0	23.2
Farm workers based on experience	371.0	65.3
Designated colleague farmer	17.0	3.0
**Dosage administered (N = 568)**		
Veterinary recommended dosage	73.0	12.9
Based on experience	449.0	79.0
Recommended by colleague farmers	46.0	8.1
**When is antibiotic administered? (N = 568)**		
Anytime animal is sick only	40.0	7.0
Farm routine practice only	13.0	2.3
Anytime animal is sick/ Farm routine practice	515.0	90.7
**Reason for antibiotic use (N = 568)**		
Prevent and treat infection	490.0	86.3
Promote growth	74.0	13.0
Prophylaxis	4	0.7
**How often do you access veterinary services (N = 210)**		
Once a month	25	11.9
1–6 months	45	21.4
Once in 6 months or more	140	66.7
**Withdrawal period (N = 568)**		
Always	93.0	16.4
Sometimes	157.0	27.6
Never	318.0	56
**Screening of manure for antibiotics presence and quantity (N = 568)**		
No	568.0	100.0

N represents total number

Only 8.5% of antibiotic administration was carried out by veterinary officers whilst, 65.3% was done by farm workers, and 23.2% by farm managers. Regarding dosage of antibiotics, 79.0% indicated they administered based on experience, 12.9% administered veterinary prescribed dosage, and 8.1% administered based on the advice from colleague farmers. Majority (90.7%) claimed they administered antibiotics as a routine practice at the farm. Of the 210 farmers who said they employed the services of veterinarian, few (11.9%) did it once in every month, 21.4% accessed between one and six months and 66.7% employed veterinary once in 6 months or more. The results of the study also showed that withdrawal period following antibiotic administration was always observed by 16.4%, and 27.6%, sometimes do it and the remaining 56.0% never observed the period. Our study found that none of the farmers screened manure for the presence of antibiotics.

### Determinants of antibiotic administration

Farmers profile, their farm characteristics and other factors that predicted the administration of antibiotics are shown in Table 3.

**Table 3:** Determinants of antibiotic administration (N = 600).

	Categories	Administered antibiotics		Crude		Adjusted	
		Yes No		OR	95% CI	OR	95% CI
Years of farming	less than 10 years	332	10	3.10	1.44–6.66	2.89	1.36–6.74
	10 years and above	236	22	1		1	
Income status^a^	High	119	1	8.22	1.11–60.8	9.10	1.14–4.16
	Low	449	31	1		1	
Level of education	Illiterate	181	4	1		1	
	Literate	387	28	3.27	1.13–9.47	4.06	2.29–10.3
Type of animal kept	Poultry	406	15	2.84	1.39–5.82	2.67	1.01–5.86
	Livestock	162	17	1		1	
Access to extension services	Yes	384	15	2.37	1.16–4.84	24.20	7.40–79.5
	No	184	17	1		1	
Registered with farmers association	Yes	351	7	5.78	2.46–13.58	4.55	1.24–9.62
	No	217	25	1		1	
Access to veterinary services	Yes	188	22	1			
	No	380	10	4.45	2.06–9.58	3.70	1.67–9.55
Location of farm	Urban	388	9	5.51	2.50–12.20	2.12	1.13–5.76
	Rural	180	23	1		1	
System of production^b^	Controlled	424	8	8.83	3.88–20.10	4.12	1.19–8.94
	Uncontrolled	144	24	1		1	
Education on antibiotic effects ^c^	Yes	154	3	3.59	1.08–11.97	3.58	1.02–12.1
	No	414	29	1		1	
Access to antibiotics	Easy	531	26	3.31	1.24–8.192	2.53	1.07–8.53
	Difficult	37	6	1		1	

^a^ income status; farmer’s annual income less than GH₵ 5,786 (low income) and greater than GH₵ 5,786 (high income)

^b^ production systems; where animals are confined to their housing permanently (controlled), or they are managed in a free range (uncontrolled).

^c^ Education on antibiotics could be formal, informal and non-formal.

Our study showed that farmers with more than 10 years of farming experience were more likely (AOR = 2.89; 95% CI, 1.36–6.74) than 10 years and less, to administer antibiotics. Then again farmers in high income bracket were highly likely (AOR = 9.10; 95% CI, 1.14–34.16) to administer antibiotics compared with farmers in the low income bracket. Level of education was shown to be a significant predictor of antibiotic administration with literate farmers highly likely (AOR = 4.25; 95% CI, 2.29–10.3) than illiterates in administering antibiotics. Type of animal kept was found to be significantly and positively related with antibiotic administration.

Farmers in the poultry industry were more likely (AOR = 2.67; 95% CI, 1.01–5.86) than other livestock farmers in administering antibiotics.

The study also revealed that farmers’ who have access to extension services have higher likelihood (AOR = 24.20; 95% CI, 7.40–79.5) than their counterparts in administering antibiotics. The results also show that registered farmers had higher odds (AOR = 4.55; 95% CI, 1.24–9.62) and therefore likely to give antibiotics to the animals. Our study showed that farmers who had access to veterinary services have higher odds (AOR = 3.70; 95% CI, 1.67–9.55) and likely to administer antibiotics compared with farmers who do not have access.

Farm location was a significant predictor where urban farms were highly likely (AOR = 2.12; 95% CI, 1.13–5.76) than rural farmers in administering antibiotics. Farmers practicing controlled production systems were more likely (AOR = 4.12, 95% CI, 1.19–8.94) to administer antibiotic than farmers in uncontrolled production system.

Farmers who had education on antibiotic effects are 260% times (AOR = 3.58 95% CI, 1.02–12.1) likely than those who had no education on antibiotics effect, to administer antibiotics. Farmers who had easy access to antibiotics were highly likely (AOR = 2.53; 95% CI, 1.07–8.53) to administer antibiotics when compared with those who had it with difficulty.

### Knowledge of farmers regarding antibiotics effect on soil ecosystem

Farmers knowledge regarding effects of antibiotics on different properties and processes of soil were inadequate with mean score ranging from 2.87 ± 0.60 and 2.98 ± 0.7 on a scale of 5.0 (Table 4), an indication that majority had inadequate knowledge of the effects of antibiotics on soil ecosystem. The lowest mean score (2.87 ± 0.60) was recorded for the statement ‘antibiotics cause the death/inhibit growth of microbial groups involved in ecosystem functions’.

**Table 4:** Knowledge of farmers regarding antibiotics effect on soil ecosystem.

Statement	Strongly Disagree	Disagree	Not sure	Agree	Strongly Agree	Mean ± Standard Deviation
Substantial quantity of antibiotic given to animals end up in manure	28 (4.70)	32 (5.30)	510 (85.00)	25 (4.20)	5 (0.80)	**2.91±0.55**
Antibiotics compete with other soil cations/anions for exchange site	23 (3.83)	41 (6.83)	516 (86.00)	6 (1.00)	14 (2.33)	**2.91±0.56**
Antibiotics cause the death/inhibit growth of microbial groups involved in ecosystem functions	15 (2.50)	8 (1.33)	563 (93.83)	12 (2.00)	2 (0.33)	**2.96±0.38**
Antibiotics influence nutrients transformation; decrease nitrification in N cycle	33 (5.50)	21 (3.50)	492 (82.00)	36 (6.00)	18 (3.00)	**2.98±0.7**
Antibiotics cause decrease in bacteria/fungi ratio in soil	13 (2.17)	12 (2.00)	557 (92.83)	11 (1.83)	7 (1.17)	**2.98±0.41**
ARGs in soil increase the likelihood of human pathogens acquiring resistance	17 (2.83)	13 (2.17)	561(93.50)	7 (1.17)	2 (0.33)	**2.94±0.40**
Antibiotics decrease soil respiration	44 (7.33)	15 (2.50)	521 (86.83)	17 (2.83)	3 (0.50)	**2.87±0.60**

Frequency and percent of participant response [number in bracket indicates percent (%)], mean score represent responses of farmers on a five point likert scale.

Similarly, mean of 2.91 ± 0.55 was obtained for ‘substantial quantity of antibiotic given to animals end up in manure’.

Moreso, mean score of (2.91±0.56), indicating inadequate knowledge was recorded for ‘antibiotics compete with other soil cations/anions for exchange site’. Similarly, mean score (2.94 ± 0.40), indicating inadequate knowledge, was obtained for the statement ‘ARGs in soil increase the likelihood of human pathogens acquiring resistance’.

Farmers’ knowledge on whether ‘antibiotics cause the death/inhibit growth of microbial groups involved in ecosystem functions’ scored a mean of 2.96 ± 0.38. Antibiotics cause decrease in bacteria/fungi ratio in soil and farmer’s mean knowledge score was 2.98 ± 0.41.

Similarly, when farmers were asked to affirm if antibiotics influence nutrients transformation; decrease nitrification in N cycle, few responded in the affirmative with mean of 2.98 ± 0.7.

## Discussion

Successful use of antibiotics in the agriculture sector for disease prevention, treatment and growth promotion have been reported [1, 2]. Regardless, about 75–80, 50–90, and 60% of the doses of tetracyclines, erythromycin, and lincomycin, respectively, are expelled in the urine and manure [27, 28]. There is however, paucity of information on the extent of use and practices regarding antibiotic administration and whether the administration of these antibiotics are associated with the farmers’ characteristics. Most importantly, the knowledge of farmers on the effect of these antibiotic on soil ecosystem remains a hypothesis in Ghana.

Practices of farmers on antibiotics utilization, showed that the purchase of antibiotics over-the-counter and without prescription was prevalent amongst farmers in the study area. This was consistent with assertions that antibiotics can easily be bought without prescription in some developing countries [29]. Even if there is prescription, it is usually invalid or issued by unlicensed veterinarians [29]. The situation is worse in Ghana because there are no antibiotic regulations for animals [30] which could have ensured antibiotics are dispensed with prescription given by a veterinarian. Buying antibiotics without prescription might result in over prescription, under prescription, buying substandard antibiotics, inappropriate administration practices including non-adherence to interdose interval, and failure to administer the right antibiotics, right dosage for a specific target pathogen. In Ghana, the involvement of veterinary services in visiting farms and administering their duties which include antibiotic utilization is very low [31] and can be attributed to low numbers of veterinarian. A ratio of 10,000 to 16,000 poultry and livestock farmers per veterinarian [32] have been reported. The high ratio results in farm managers and workers relying on experience often associated with familiarity with symptoms of the disease, to purchase and administer antibiotics. These occurrences could lead to the abuse of antibiotics and consequently serve as a catalyst for antibiotic gene resistance development and spread. Wrong diagnoses could also expose healthy animals to avoidable antibiotics intake predisposing commensal microbes to resistant development over time [33, 34]. One recommended healthy practice of antibiotic utilization is by observing withdrawal period following antibiotic administration. This was virtually absent which implies that retention of antibiotics in the meat and residues in the manure is unavoidable.

Our study also revealed that, none of the farmers ever screened their manure for antibiotic residue. A number of studies have reported substantial quantities of antibiotic in manure [9, 10] posing a threat to the environment. Manure is a major soil nutrient source for farmers in Ghana and it is obvious that farmers might have introduced antibiotic into the environment through the application of manure to agricultural field.

A number of factors were also found to influence the administration of antibiotics which included farmers’ characteristics and related variables. It was evident that as years of farming increased, farmer’s experiences with new technologies also increased [35]. This is an indication that the exposure of old farmers to negative effect of antibiotics might have influenced their decision not to give antibiotics to their animals. It has been espoused that most old farmers believe in their own experienced and get little involved in new technologies [35]. In contrast, young farmers in their quest to succeed and due to their inexperience with disease management may heavily depend on antibiotics unlike the aged who may employ integrated management which seeks minimal antibiotic inputs.

It is imperative that the provision of education to farmers would help their analysis of information and enhance their knowledge from reading labels and other sources of information. It would ensure administration of appropriate antibiotics for a specific disease condition, ensure precision in dosage and observation of withdrawal period. From our study, appreciable number of farmers could not read and write and therefore may be handicapped in processing and analyzing information on antibiotics. This consequently, results in inappropriate handling and administration of the antibiotics [32]. Farmers income was an important variable that influenced administration of antibiotics to farmers. The income level of farmers affects what they can afford, therefore increased use of antibiotics amongst farmers is associated with their affordability within the high income earners.

Furthermore, other variables such as the extent of susceptibility of the type of animal to infection had association with antibiotic administration. It has been reported that poultry are associated with higher infections from *Salmonella species* and *Escherichia*. *coli* [36] and this explains why majority of farmers raising poultry used antibiotics. It is evident that increased access to extension services has direct relationship with antibiotics administration to animals. Farmers exposed to extension services may have been provided with education and training on the need to use prescribed antibiotics [37].

Similarly, veterinary officers have key roles in the poultry and livestock sector and this include but not limited to educating and training of farmers on best practices including diagnosing disease conditions of the animals, prescribing and administration of drugs. The exposure of farmers to these veterinarians enhances farmers’ knowledge and acceptance to give antibiotics to their animals. Empirically, the location of the farm has a bearing on antibiotic administration such that rural farmers may be less privileged in terms of unavailability of appropriate antibiotics, veterinarians, high cost. Moreover, most rural farmers might not have the financial strength to travel to urban centers to purchase the antibiotics. The counterparts in the urban setting are advantaged in accessing information on antibiotics, easy access to antibiotics, technical support and availability of veterinarians.

Characteristically, most of our farmers did not belong to farmers’ groups. Belonging to farmers’ association helps to access interventions which include education on products needed to facilitate farm operations and enhance productivity. The association also promotes internal learning where farmers learn from each other [38]. In other instances, farmers invite key stakeholders, such as veterinary officers to provide technical advice on the diagnosis and treatment of animal diseases. Through these training farmers are able to learn to treat their own sick animals or consult other farmers who are experienced, cutting down cost involved in engaging veterinary doctor.

Where animals are kept in a controlled system, farmers gave antibiotics more than farmers in uncontrolled (free range) production system did. These could be as a result of the routine practice of antibiotic administration usually through feed or water given to the animals, usually in the intensive system. Unlike the intensive system, animals raised under free range system has disadvantage of not been fed regularly with antibiotic laden feed or water, so could be susceptible to disease pathogens acquired as they move from one location to the other.

Education of farmers regarding antibiotics could be pivotal in influencing farmers’ decision to administer antibiotics. Less education means, farmers are bereft of information on antibiotics, hence there is likelihood of compromised practices. Education on antibiotics could come in the form of seminars, workshops and farm visits to increase the understanding of farmers on antibiotics, expose farmers to handling, administering and observation of withdrawal period. Our study corroborates other assertions that access to antibiotics was easy [39] in most developing countries which has the propensity to increase antibiotic administration. It would interest the scientific world to know that in some of these developing countries including Ghana, antibiotics are sold without need to go through checks, such as official prescription from a veterinarian. This calls for stringent measures to regulate the supply, purchasing and use of antibiotic in Ghana.

A major source of plant nutrient is manure, but have been found to contain substantial amounts of antibiotic residues [40, 41, 42]. However, the knowledge of stakeholders especially farmers on the possible effect on the soil ecosystem has received little attention. Several studies have indicated negative interaction of antibiotic contaminated manure on the soil ecosystem [8, 43] hence it will be plausible to increase the farmers’ knowledge in this regard. Adequate knowledge on antibiotics will inform and shape the practices of farmers regarding antibiotics, consequently reducing risk to human and soil ecosystem. Our study, revealed that farmers were bereft of knowledge regarding antibiotic effect on soil properties and processes. Some effects for which farmers showed inadequate knowledge were; substantial quantity of antibiotic given to animals end up in manure [9, 10], antibiotics cause the death/inhibit growth of microbial groups involved in ecosystem functions [44], antibiotics affect bacteria/fungi ratio in soil [8], high antibiotic rate decrease soil respiration/catabolism’ [45], high antibiotic rate influence nutrients transformation and decrease nitrification in nitrogen cycle [21], Other effects considered with inadequate knowledge were; antibiotics compete with other soil cations/anions for exchange site [22, 46], which could lead to enhanced mobility and possible leaching of essential soil macro nutrients such as Mg^2+^ and Ca^2+^, increase ARGs in soil [12, 13]. The low level of knowledge on the effect of antibiotic laden manure will perpetuate resistance development and negatively influence soil quality and food security.

It is important to note that our research did have limitations. This study sampled respondents from five districts each from the regions with assumption that; the selected districts were amongst leading poultry and livestock producers. This might have resulted in leaving out possible districts with high level of production. Although this couldn’t compromise our findings due to the census method used. Moreover, to be able to get the true reflection of the phenomena under investigation, the study used census method to ensure data is captured from all farmers who raised birds and livestock. Generalizing the findings of our study to reflect the state of antibiotic utilization and knowledge of farmers regarding antibiotics effects on soil in Ghana, is valid. However, regions in Northern Ghana where farmers are mostly into diary production and reduced poultry could report contrasting findings compared with our findings. This calls for research study in Northern Ghana. Irrespective of the limitations, our study, the first of its kind in Ghana was able reveal the knowledge of farmers regarding antibiotic effects on soil ecosystem, is an important finding to aid in policy directions for antibiotic deployment in the animal production sector and use of manure for soil fertility management.

## Conclusion

In conclusion, our study revealed high and indiscriminate utilization of antibiotics amongst animal producers in Ghana. It also exposed the poor practices regarding antibiotic use and the inadequate knowledge regarding its effect on soil ecosystem amongst farmers in Ghana. This calls for development of strategies to increase awareness on antibiotics amongst farmers because its misuse can negatively impact human, animals, environment and food security. We also suggest a collaboration between Ghana Health Service, Food and Drugs Authority and Ministry of Food and Agriculture to draft and implement regulations to guide the use of antibiotics at the farm gate.

## Supporting information

S1 File Questionnaire on farmer’s utilization of antibiotics. (PDF)

## References

[1] De Briyne N, Atkinson J, Pokludová L, Borriello SP. Antibiotics used most commonly to treat animals in Europe. The Veterinary Record. 2014 10 4;175(13):325 10.1136/vr.102462 24899065 PMC4215272

[2] Mathew AG, Liamthong S, Lin J, Hong Y. Evidence of class 1 integron transfer between *Escherichia coli* and *Salmonella spp*. on livestock farms. Foodborne Pathogens and Disease. 2009 10 1;6(8):959–64. 10.1089/fpd.2009.0263 19630513

[3] Castanon JI. History of the use of antibiotic as growth promoters in European poultry feeds. Poultry science. 2007 11 1;86(11):2466–71. 10.3382/ps.2007-00249 17954599

[4] Stockwell VO, Duffy B. Use of antibiotics in plant agriculture. Revue Scientifique Et Technique-Office International Des Epizooties. 2012;31(1). 10.20506/rst.31.1.2104 22849276

[5] Hu X, Zhou Q, Luo Y. Occurrence and source analysis of typical veterinary antibiotics in manure, soil, vegetables and groundwater from organic vegetable bases, northern China. Environmental Pollution. 2010 9 1;158(9):2992–8. 10.1016/j.envpol.2010.05.023 20580472

[6] Migliore L, Cozzolino S, Fiori M. Phytotoxicity to and uptake of enrofloxacin in crop plants. Chemosphere. 2003 8 1;52(7):1233–44. 10.1016/S0045-6535(03)00272-8 12821004

[7] Msoffe PL, Aning KG, Byarugaba DK, Mbuthia PG, Sourou S, Cardona C, et al Handbook of poultry diseases important in Africa. A Project of the Global Livestock CRSP pp. 2009;83.

[8] Cycoń M, Mrozik A, Piotrowska-Seget Z. Antibiotics in the Soil Environment—Degradation and their Impact on Microbial Activity and Diversity. Frontiers in microbiology. 2019;10. 10.3389/fmicb.2019.00338 PMC6418018 30906284

[9] Hou J, Wan W, Mao D, Wang C, Mu Q, Qin S, et al Occurrence and distribution of sulfonamides, tetracyclines, quinolones, macrolides, and nitrofurans in livestock manure and amended soils of Northern China. Environmental Science and Pollution Research. 2015 3 1;22(6):4545–54. 10.1007/s11356-014-3632-y 25318415

[10] DeVries SL, Zhang P. Antibiotics and the terrestrial nitrogen cycle: a review. Current Pollution Reports. 2016 3 1;2(1):51–67.

[11] Massé D, Saady N, Gilbert Y. Potential of biological processes to eliminate antibiotics in livestock manure: an overview. Animals. 2014 6;4(2):146–63. 10.3390/ani4020146 26480034 PMC4494381

[12] Su JQ, Wei B, Xu CY, Qiao M, Zhu YG. Functional metagenomic characterization of antibiotic resistance genes in agricultural soils from China. Environment international. 2014 4 1;65:9–15. 10.1016/j.envint.2013.12.010 24412260

[13] Kyselková M, Kotrbová L, Bhumibhamon G, Chroňáková A, Jirout J, Vrchotová N, et al Tetracycline resistance genes persist in soil amended with cattle feces independently from chlortetracycline selection pressure. Soil Biology and Biochemistry. 2015 2 1;81:259–65.

[14] WHO (World Health Organization). 2014 Antimicrobial Resistance: Global Report on Surveillance Geneva: WHO.

[15] Al-Emran HM, Eibach D, Krumkamp R, Ali M, Baker S, Biggs HM, et al Multi country molecular analysis of *Salmonella enterica serovar Typhi* with reduced susceptibility to ciprofloxacin in sub-Saharan Africa. Clinical Infectious Diseases. 2016 3 1;62(suppl_1):S42–6. 26933020 10.1093/cid/civ788 PMC4772832

[16] Marquardt RR, Li S. Antimicrobial resistance in livestock: advances and alternatives to antibiotics. Animal Frontiers. 2018 4 19;8(2):30–7. 10.1093/af/vfy001 PMC6951930 32002216

[17] Akimenko YV, Kazeev KS, Kolesnikov SI. Impact assessment of soil contamination with antibiotics (For example, an ordinary chernozem). American Journal of Applied Sciences. 2015 2 1;12(2):80.

[18] Xu Y, Yu W, Ma Q, Wang J, Zhou H, Jiang C. The combined effect of sulfadiazine and copper on soil microbial activity and community structure. Ecotoxicology and environmental safety. 2016 12 1;134:43–52. 10.1016/j.ecoenv.2016.06.041 27584823

[19] Wei X, Wu SC, Nie XP, Yediler A, Wong MH. The effects of residual tetracycline on soil enzymatic activities and plant growth. Journal of Environmental Science and Health, Part B. 2009 7 17;44(5):461–71. 10.1080/03601230902935139 20183051

[20] Toth JD, Feng Y, Dou Z. Veterinary antibiotics at environmentally relevant concentrations inhibit soil iron reduction and nitrification. Soil Biology and Biochemistry. 2011 12 1;43(12):2470–2.

[21] Rosendahl I, Siemens J, Kindler R, Groeneweg J, Zimmermann J, Czerwinski S, et al Persistence of the fluoroquinolone antibiotic difloxacin in soil and lacking effects on nitrogen turnover. Journal of environmental quality. 2012 7 1;41(4):1275–83. 10.2134/jeq2011.0459 22751072

[22] Wegst-Uhrich SR, Navarro DA, Zimmerman L, Aga DS. Assessing antibiotic sorption in soil: a literature review and new case studies on sulfonamides and macrolides. Chemistry Central Journal. 2014 12;8(1):5 10.1186/1752-153X-8-5 24438473 PMC3905979

[23] WHO guidelines on use of medically important antimicrobials in food-producing animals Geneva: World Health Organization; 2017. Licence: CC BY-NC-SA 3.0 IGO. 29578662

[24] Darko G, Borquaye LS, Acheampong A, Oppong K. Veterinary antibiotics in dairy products from Kumasi, Ghana. Cogent Chemistry. 2017 1 1;3(1):1343636.

[25] Osei Sekyere J. Antibiotic types and handling practices in disease management among pig farms in Ashanti Region, Ghana. Journal of veterinary medicine. 2014;2014. 10.1155/2014/531952 PMC4590843 26464936

[26] Armar-Klemesu M, Maxwell D. Accra: Urban agriculture as an asset strategy, supplementing income and diets. Growing cities, growing food. Urban agriculture on the policy agenda. 2000 4:183–208.

[27] Kumaranayake L, Lake S, Mujinja P, Hongoro C, Mpembeni R. How do countries regulate the health sector? Evidence from Tanzania and Zimbabwe. Health policy and planning. 2000 12 1;15(4):357–67. 10.1093/heapol/15.4.357 11124238

[28] Sarmah AK, Meyer MT, Boxall AB. A global perspective on the use, sales, exposure pathways, occurrence, fate and effects of veterinary antibiotics (VAs) in the environment. Chemosphere. 2006 10 1;65(5):725–59. 10.1016/j.chemosphere.2006.03.026 16677683

[29] Sommanustweechai A, Chanvatik S, Sermsinsiri V, Sivilaikul S, Patcharanarumol W, Yeung S, et al Antibiotic distribution channels in Thailand: results of key-informant interviews, reviews of drug regulations and database searches. Bulletin of the World Health Organization. 2018 2 1;96(2):101 10.2471/BLT.17.199679 29403113 PMC5791780

[30] Yevutsey SK, Buabeng KO, Aikins M, Anto BP, Biritwum RB, Frimodt-Møller N, et al Situational analysis of antibiotic use and resistance in Ghana: Policy and regulation. BMC public health. 2017 12;17(1):896 10.1186/s12889-017-4910-7 29169340 PMC5701378

[31] Adams F, Ohene-Yankyera K. Determinants of small ruminant farmers decision to participate in veterinary services in Northern Ghana. Journal of Veterinary Medicine and Animal Health. 2015 5 31;7(5):193–204.

[32] Turkson PK. Profile of veterinarians and veterinary practice in Ghana. Tropical animal health and production. 2003 8 1;35(4):321–40. 10.1023/a:1025141321334 14509539

[33] Capita R, Riesco-Peláez F, Alonso-Hernando A, Alonso-Calleja C. Exposure of Escherichia coli ATCC 12806 to sublethal concentrations of food-grade biocides influences its ability to form biofilm, resistance to antimicrobials, and ultrastructure. Appl. Environ. Microbiol. 2014 2 15;80(4):1268–80. 10.1128/AEM.02283-13 24317080 PMC3911067

[34] van Bijnen EM, Paget J, de Lange-de Klerk ES, den Heijer CD, Versporten A, Stobberingh EE, et al Collaboration with the APRES Study Team. Antibiotic exposure and other risk factors for antimicrobial resistance in nasal commensal *Staphylococcus aureus*: an ecological study in 8 European countries. PLoS One. 2015 8 11;10(8):e0135094 10.1371/journal.pone.0135094 26262679 PMC4532423

[35] Ransom JK, Paudyal K, Adhikari K. Adoption of improved maize varieties in the hills of Nepal. Agricultural economics. 2003 12;29(3):299–305.

[36] Agyare C, Boamah VE, Zumbi CN, Osei FB. Antibiotic use in animal production and its effects on bacterial resistance. 10.5772/intechopen.79371

[37] Chauhan AS, George MS, Chatterjee P, Lindahl J, Grace D, Kakkar M. The social biography of antibiotic use in smallholder dairy farms in India. Antimicrobial Resistance & Infection Control. 2018 12;7(1):60. 29744041 10.1186/s13756-018-0354-9 PMC5930822

[38] Conley TG, Udry CR. Learning about a new technology: pineapple growers in Ghana. Preprint, Northwestern University. 2000. Available from www.econ.yale.edu/~udry/pdf/aercycle3.pdf

[39] Boamah VE, Agyare C, Odoi H, Dalsgaard A. Practices and factors influencing the use of antibiotics in selected poultry farms in Ghana.

[40] Leal RM, Figueira RF, Tornisielo VL, Regitano JB. Occurrence and sorption of fluoroquinolones in poultry litters and soils from São Paulo State, Brazil. Science of the total Environment. 2012 8 15;432:344–9. 10.1016/j.scitotenv.2012.06.002 22750180

[41] Li YX, Zhang XL, Li W, Lu XF, Liu B, Wang J. The residues and environmental risks of multiple veterinary antibiotics in animal faeces. Environmental monitoring and assessment. 2013 3 1;185(3):2211–20. 10.1007/s10661-012-2702-1 22692716

[42] Dahshan H, Abd-Elall AM, Megahed AM, Abd-El-Kader MA, Nabawy EE. Veterinary antibiotic resistance, residues, and ecological risks in environmental samples obtained from poultry farms, Egypt. Environmental monitoring and assessment. 2015 2 1;187(2):2 10.1007/s10661-014-4218-3 25600402

[43] Pornsukarom S, Thakur S. Assessing the impact of manure application in commercial swine farms on the transmission of antimicrobial resistant *Salmonella* in the environment. PloS one. 2016 10 18;11(10):e0164621 10.1371/journal.pone.0164621 27755598 PMC5068702

[44] Marx MC, Kandeler E, Wood M, Wermbter N, Jarvis SC. Exploring the enzymatic landscape: distribution and kinetics of hydrolytic enzymes in soil particle-size fractions. Soil Biology and Biochemistry. 2005 1 1;37(1):35–48.

[45] Liu F, Ying GG, Tao R, Zhao JL, Yang JF, Zhao LF. Effects of six selected antibiotics on plant growth and soil microbial and enzymatic activities. Environmental Pollution. 2009 5 1;157(5):1636–42. 10.1016/j.envpol.2008.12.021 19157661

[46] Call JJ. Cation Exchange Processes Involving the Agricultural Antibiotic Tylosin in Soil and Soil Minerals.

