# Supplementary material for: Antibiotics utilization and farmers’ knowledge of its effects on soil ecosystem in the coastal drylands of Ghana
Source: PLoS One. 2020 Feb 6;15(2):e0228777. doi: 10.1371/journal.pone.0228777 (PMC7004350; doi:10.1371/journal.pone.0228777)
Supplement: S1 File — (PDF) [file pone.0228777.s001.pdf]

DEPARTMENT OF SOIL SCIENCE

SCHOOL OF AGRICULTURE

UNIVERSITY OF CAPE COAST

This questionnaire seeks to ascertain the extent of antibiotic use amongst farmers in the animal sector. Please be assured that your privacy is protected and you may withdraw from the study at any time.

1. Indicate your sex
   a. male { } b. Female { }
2. What is your age?
   a. < 30 { } b. 30 years and more { }
3. Marital status
   a. Married { } b. Unmarried { }
4. Years of farming?
   a. Less than ten years { } b. 10 years and above { }
5. What's your annual income from the farm
   a. Less than GH₵ 5,786 (low income) { } b. above GH₵ 5,786 (high income) { }
6. What is your level of education?
   a. Literate { }b. Illiterate { }
7. What type of animal do you keep?
   a. Poultry { } b. livestock { }
8. Veterinary personnel availability
   a. always { } b. sometimes { } c. not at all { }
9. Do you have access to extension services?
   a. Yes { } b. No { }
10. Are you registered with any farmer group?
    a. Yes { } b. No {]
11. Have you ever employed the services of veterinary officer?
    a. Yes { } b. No { }
12. What's the location of the farm?
    a. Urban { } b. Peri Urban/Rural { }
13. Under which production system do you keep your animals?
    a. Controlled system{ } b. Uncontrolled (free range) system { }
14. Have you ever received education on antibiotics (either formal, non formal and informal)? a. Yes { } b. No { }
15. How difficult is it to have access to antibiotics?

a. Easy { } b. Difficult { }

16. Have you ever administered antibiotics to your animals?
    a. Yes { } b. No
17. If Yes to Question 16, how do you access the antibiotics?
    a. Purchased with veterinary prescription { }
    b. Purchased over the counter without prescription { }
    c. Colleague farmer without prescription { }
    d. Agro dealer without prescription { }
18. Who administered antibiotics to the animals?
    a. Veterinary officer { } b. farm manager { } c. farm owner { } d. other, specify { }……………………………………………….
19. What dosage was administered?
    a. Veterinary recommended dosage { }
    b. Dosage administered based on experience { }
    c. Based on a colleague farmer recommendation { }
20. When do you administer antibiotics
    a. Anytime animal is sick{ }
    b. Routine farm practice { }
    c. Anytime animal is sick/Farm routine practice { }
21. What was your reason for using antibiotics?
    a. Prevent and treat infections { } b. Promote growth { } c. Prophylaxis { }
22. If you employed the services of veterinarian (question 11) please indicate how often you do that.
    a. Once a month { }
    b. 1 – 6 months { }
    c. Once in 6 months or more { }
23. How often do you observe withdrawal period?
    a. Always { }
    b. Sometimes { }
    c. Never { }
24. Have you ever screened your antibiotics for the presence and concentration of antibiotics?
    a. Yes { } b. No { }

Knowledge regarding antibiotic effect on soil ecosystem

Indicate your level of agreement with the following items (25 – 31) about antibiotics effect on the soil.

Statements	Strongly Disagree	Disagree	Not sure	Agree	Strongly Agree
25. Substantial quantity of antibiotics given to animals end up in manure					
26. Antibiotics compete with other soil cations/anions for exchange site					
27. Antibiotics cause the death/inhibit growth of microbial groups involved in ecosystem functions					
28. Antibiotics influence nutrients transformation; decrease nitrification in N cycle					
29. Antibiotics cause decrease in bacteria/fungi ratio in soil					
30. Antibiotic Resistance Genes (ARGs) in soil increase the likelihood of human pathogens acquiring resistance					
31. Antibiotics decrease soil respiration					
